# Anchoring structure of the calvarial periosteum revealed by focused ion beam/scanning electron microscope tomography

**DOI:** 10.1038/srep17511

**Published:** 2015-12-02

**Authors:** Shingo Hirashima, Keisuke Ohta, Tomonoshin Kanazawa, Kei-ichiro Uemura, Akinobu Togo, Munetake Yoshitomi, Satoko Okayama, Jingo Kusukawa, Kei-ichiro Nakamura

**Affiliations:** 1 Division of Microscopic and Developmental Anatomy, Department of Anatomy, Kurume University School of Medicine, Kurume, 830-0011, Japan; 2 Dental and Oral Medical Center, Kurume University School of Medicine, Kurume, 830-0011, Japan; 3 Electron Microscopic Laboratory, Central Research Unit of Kurume University, Kurume, 830-0011, Japan

## Abstract

An important consideration in regeneration therapy is the fact that the tissue surrounding an organ supports its function. Understanding the structure of the periosteum can contribute to more effective bone regeneration therapy. As a cellular source, the periosteum also assists bone growth and fracture healing; this further necessitates its direct contact with the bone. However, its anchoring strength appears to be inexplicably stronger than expected. In this study, we used focused ion beam/scanning electron microscope tomography to investigate ultrathin serial sections as well as the three dimensional ultrastructure of the periosteum to clarify the architecture of its anchoring strength, as such assessments are challenging using conventional methods. We discovered perforating fibres that arise from the bone surface at 30 degree angles. Additionally, the fibres across the osteoblast layer were frequently interconnected to form a net-like structure. Fibroblast processes were observed extending into the perforating fibres; their morphologies were distinct from those of typical fibroblasts. Thus, our study revealed novel ultrastructures of the periosteum that support anchorage and serve as a cellular source as well as a mechanical stress transmitter.

In recent years, numerous basic studies and clinical applications describing the periosteum as a cellular source have been reported^1^,^2^,^3^,^4^,^5^. The periosteum is an essential tissue that maintains bone environment and function. Because organ functions involve biological cooperation with the surrounding tissues and other organs^6^,^7^, understanding the proper structure of the periosteum could potentially contribute to developing more efficacious bone regeneration modalities.

The periosteum consists of two layers, outer fibrous and inner cambial. The cambial layer includes stem cells, osteoprogenitor cells, and osteoblasts, all of which influence bone growth^8^ and fracture healing^9^,^10^,^11^. As the periosteum also functions as a source for cells, it must resist mechanical stress while suspended *in situ*. In fact, soft tissue does not separate from the bone when exposed to usual external forces except in the event of high-energy trauma.

The periosteum attaches to the bone via distinct collagen bundles, which are perforating fibres, that connect calcified bone matrices and other organs. Although these fibres are sometime referred as Sharpey’s fibres, this term is much more frequently applied to connections between hard tissues such as the periodontal ligament and skeletal sutures^12^,^13^,^14^,^15^. In the case of flattened bones like the parietal bone, the fibres connect the bone to its surrounding fibrous tissue. Because the details of the structural organization of the point where the connecting fibre meets the fibrous layer are unclear, we focused on the ultrastructural architecture of these distinct fibres, which we refer to as “perforating fibres” in this report.

In periosteum, there are two physical anchoring mechanisms. One involves extending cellular processes that make contact between osteocytes or osteoblast, and are referred to as the lacuno-canalicular network. The other involves perforating fibres. These fibres are considered simple nail-like structures that connect the fibrous layer of the periosteum. These structures exert anchoring strength; however, the reason for this is unclear.

Many studies have revealed the structures of the periosteum and perforating fibres. Light microscopy showed that a perforating fibre has a diameter of roughly 8–25 μm^16^. The overall structure of perforating fibres in bone matrix can be observed by fluorescent and polarized light microscopy;^16^ however, the structure outside of the bone matrix is quite difficult to analyse using light microscopy because of the large amounts of collagen bundles in the outer fibrous layer. Transmission electron microscopy has sufficient resolution to observe the detail bundle structure, but there have been no reports describing their three-dimensional (3D) organization using the serial sectioning method. Scanning electron microscopy revealed the distribution pattern of the perforations on bone surfaces in chemically or physiologically separated specimen surfaces of bone and periosteum. In order to understand the nature of the periosteum/bone junction, as well as the strength of the periosteum anchoring mechanism, it is necessary to observe the intact periosteum while attached to the bone with an electron microscope.

Recently, a new method for serial sectioning as well as 3D analytical scanning electron microscopy, namely focused ion beam/scanning electron microscope tomography (FIB/SEM tomography), has been developed^17^,^18^,^19^. FIB/SEM tomography is a novel approach: A specimen is milled with a focused ion beam, and the new block face is imaged with SEM. This process is performed repetitively and automatically for a set number of times and is called the “Slice and View” method. It enables observation of hundreds of serial sections and allows 3D structural analysis of tissue with areas ranging from 5 × 5 × 5 μm to 100 × 100 × 100 μm with high resolution. Additionally, it is possible to reconstruct 3D structures and quantitatively analyse them. We previously used FIB/SEM tomography to investigate the structure of organelles and cell morphology^19^,^20^,^21^.

In this study, we investigated the 3D structure of the calvarial periosteum by ultrathin serial sections, and analysed the relationship between the perforating fibres and their associated cells.

## Methods

All experiments were performed in accordance with the National Institutes of Health Guidelines for animal research. All animal procedures were approved by the Board for Animal Experiments of Kurume University.

### Preparation for light microscopy specimens

Fifteen-week-old male Sprague-Dawley rats (n = 3) were deeply anesthetized with diethyl ether and sodium pentobarbital (50 mg/kg), and were transcardially perfused through the left ventricle with heparin containing (10 U/mL) saline, followed by fixing with 4% paraformaldehyde in PBS. After perfusion, parietal bones containing periosteum were removed from the skull using a saw. The specimens were immersed in the same fixative for 2 h at 4 °C. They were then each rinsed in buffer and decalcified in 10% ethylenediaminetetraacetate (EDTA) solution for 3–4 weeks. EDTA solution was exchanged every 3 days. Afterwards, specimens were trimmed, washed 3 times for 5 min in PBS, and immersed in PBS containing 30% sucrose for 3 days at 4 °C, after which they were frozen in Tissue-Tek® OCT compound (Sakura Finetek, USA). Frozen blocks were cut into 6-μm-thick sections with CryoMicrotome, CM1950 (Leica, Germany). The sections were stained with haematoxylin and eosin and observed.

### Preparation of electron microscopy specimens

Specimens were prepared as described previously^19^. Fifteen-week-old male Sprague-Dawley rats (n = 3) were deeply anesthetized with diethyl ether and sodium pentobarbital (50 mg/kg), and were transcardially perfused through the left ventricle with heparin containing (10 U/ml) saline, followed by fixing with 2% paraformaldehyde, and placing in 2.5% glutaraldehyde in 0.1 M cacodylate (pH 7.3) buffer for electron microscopy. After perfusion, parietal bones with periosteum were removed from the skull using a saw. The specimens were immersed in the same fixative for 2 h at 4 °C. They were each rinsed in buffer and decalcified as described above. After decalcification, the specimens were rinsed in buffer 3 times for 10 min each.

Next, specimens were cut into small cubes and were subjected to a post-fixation and *en bloc* stain procedure as follows: After 3 washes in the cacodylate buffer, the specimens were post-fixed for 2 h in a solution containing 2% osmium tetraoxide and 1.5% potassium ferrocyanide in the cacodylate buffer at 4 °C. The specimens were then washed 3 times with distilled water and immersed in 1% thiocarbohydrazide solution for 1 h. After 5 washes with distilled water, they were further immersed in 2% osmium tetraoxide in distilled water and washed 3 times with distilled water. The specimens were then stained *en bloc* in a solution of 4% uranyl acetate dissolved in a 25% methanol solution overnight for contrast enhancement, and were washed with distilled water. The specimens were then further stained by Walton’s lead aspartate solution for 1 h^22^. Afterwards, they were dehydrated in an ethanol series (25%, 50%, 70%, 80%, 90% and twice in 100% for 10 min each), followed by infiltration with epoxy resin (Epon 812, TAAB, England) mixture, and polymerization for 72 h at 60 °C. The surfaces of the embedded specimens were exposed using a diamond knife on an Ultracut E microtome (Leica, Germany). The resin blocks were then trimmed down and placed on a holder.

### FIB/SEM tomography and 3D-structure reconstruction

**Figure 1:**
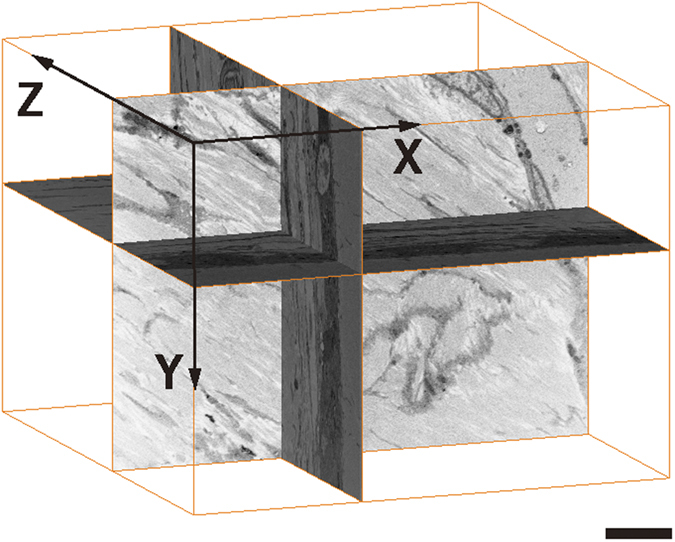
Structural analysis of the priosteum by focused ion beam/scanning electron microscope (FIB/SEM) tomography. Data with FIB/SEM tomography is analysed using image analysis software (Amira 5.5.0). Shown are the X-Y-Z plane sections. Bar scale, 7 μm.

Serial images of the block face were acquired by repeated cycles of sample surface milling and imaging using the Slice & View G2 operating software (FEI). The milling was performed with a gallium ion beam at 30 kV with a current of 15 nA. The milling pitch was set to 100 nm/step and 600 cycles. The images were acquired at a landing energy of 5.5 keV. Additional acquisition parameters were as follows: beam current = 100 pA, dwell time = 10 μs/pixel, image size = 1024 × 883 pixels (59 μm × 51 μm), and pixel size = 58 nm/pixel. Under the conditions of our study, “Slice and View” process took about 8 hours. The resultant image stack, segmentation, and 3D reconstruction were processed using open software from Fiji (http://fiji.sc/Fiji), and Amira 5.5.0 software (FEI Visualization Science Group, Burlington, MA). Images could be observed with optional X-Y-Z plane sectioning (Fig. 1). After reconstruction, the angle at which the perforating fibres intersect with the bone surface was measured.

With the Amira software, any section containing the vector of perforating fibre and bone surface were obtained. Each of the normal vectors was then calculated. To measure the angle of the perforating fibre at the bone surface, the inner product between the normal vectors of the perforating fibre and the bone surface was calculated.

## Results

### Light Microscope

**Figure 2:**
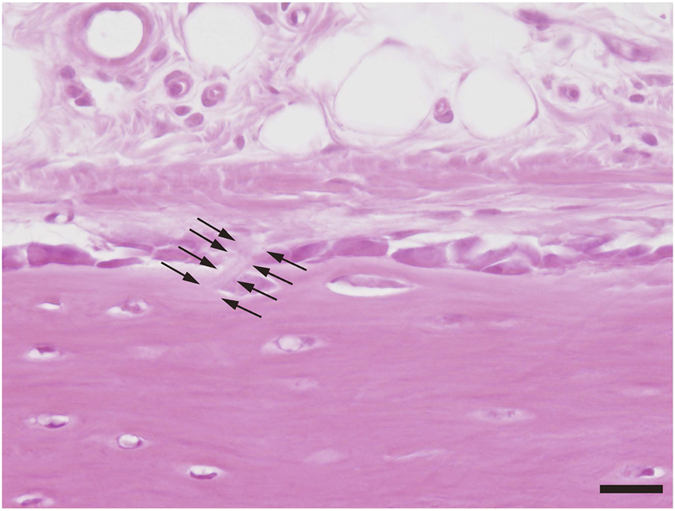
Perforating fibre observed under conventional plain light microscopy. A dense collagen fibre (arrows) is visible in the cambial layer in this decalcified 6-μm-thick section. However, the perforating fibre cannot be traced to the fibrous layer and bone. Bar scale, 20 μm.

Dense collagen bundles were detected perforating the cambial layer (Fig. 2). Collagen bundles were not observed in the fibrous layer of the periosteum. Furthermore, both ends of the collagen bundles were indistinct. Other structures were not observable by light microscopy.

### FIB/SEM tomography: serial cross-section

**Figure 3:**
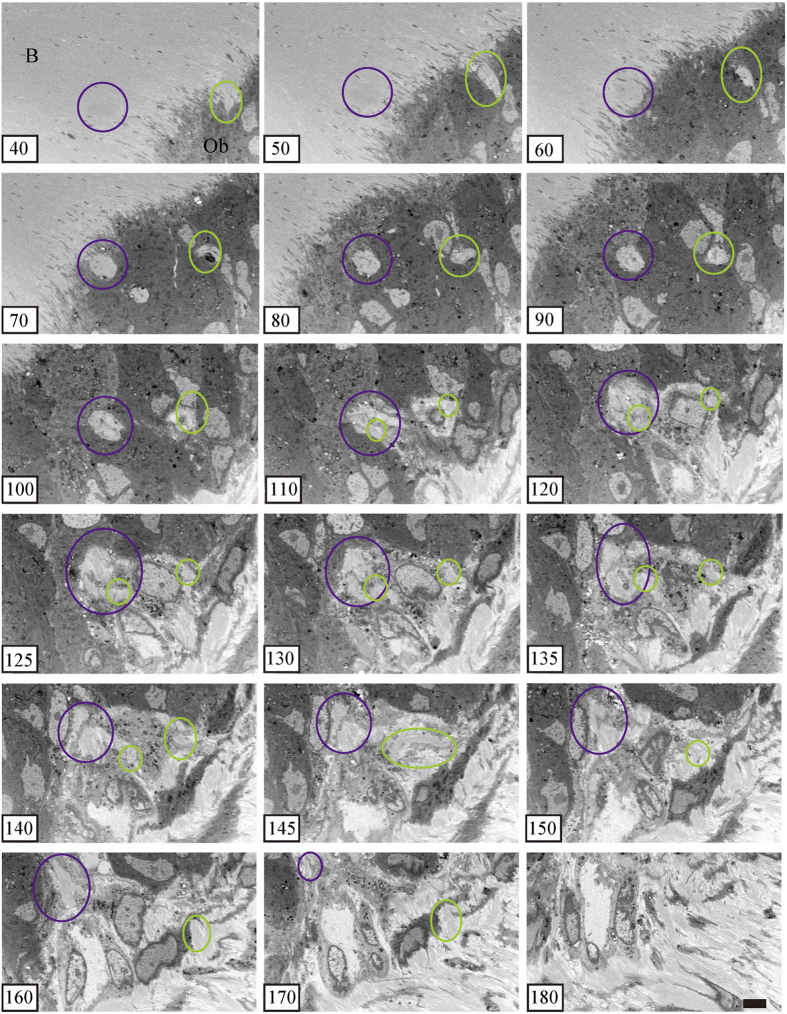
Serial section of the X-Y plane. Two perforating fibres (purple and green circles) perforate the bone (slices 40–90) and connect to each other in the fibrous layer (slices 100–170). The ultrastructure of the perforating fibre can be observed in detail with focused ion beam/scanning electron microscope tomography. B, bone. Ob, osteoblast. Bar scale, 5 μm for all sections.

**Figure 4:**
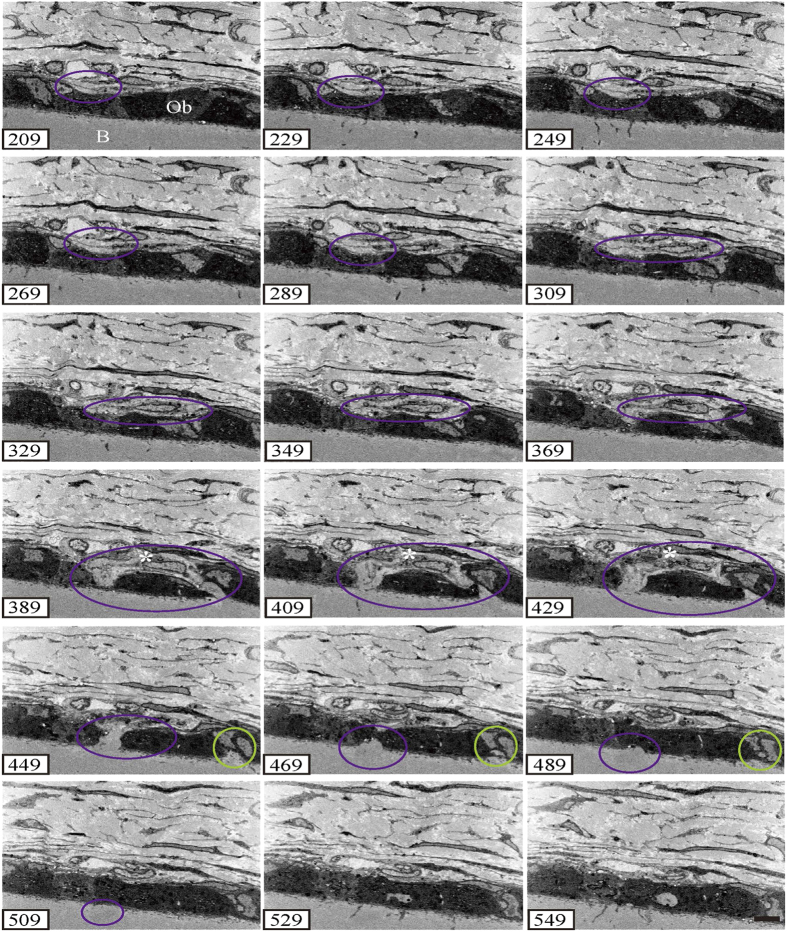
Serial section of the X-Z plane. Two perforating fibres (purple and green circles) are connected to each other in the fibrous layer. The highlighted fibres correspond to those in Fig. 3. The cell process extends along the perforating fibre (arrows). The asterisks (*) correspond to the same as those in Figs 5,8. B, bone. Ob, osteoblast. Bar scale, 5 μm for all sections

In serial cross-sectioning with FIB/SEM tomography, the collagen bundle of the perforating fibre passes between the cambial layers and perforates the bone at the periosteum (Figs 3, 4). Compared to the collagen bundle of the fibrous layer, the perforating fibre is featureless. In the fibrous layer, the perforating fibre was clearly visible among the network of other fibres. We observed two perforating fibres connected to each other in the fibrous layer (Fig. 3, purple and green circles, slice numbers 100–170; Fig. 4, purple and green circles, slice numbers 429–489) and perforated the bone (Fig. 3 slice numbers 40–90). The ultrastructure of the perforating fibre can be observed in detail (Figs 3, 4).

**Figure 5:**
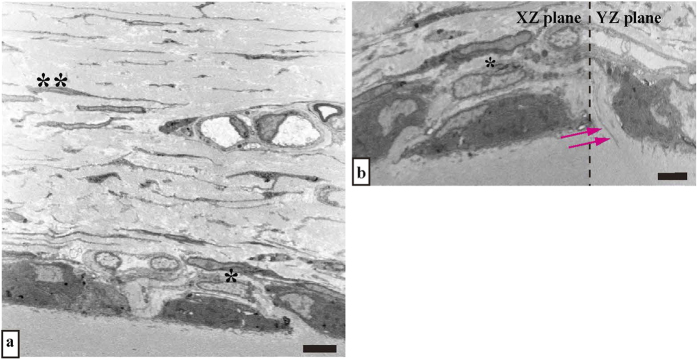
Two types of fibroblasts in the periosteum. (**a**) Typical fibroblasts (**) are observed. (**b**) The fibroblast processes extend to the perforating fibre (*). The process spreads to the bone surface (magenta arrows). Bar scale, 5 μm in both panels.

Typical fibroblasts were observed in the middle of the fibrous layer (Fig. 5a, denoted by **). In the periosteum, these fibroblasts are flattened with oval nuclei, spindle shapes, and smooth surfaces. Some fibroblasts were located along the lines of the perforating fibre in the periosteum (denoted by * in Fig. 4, slices 329–429; and Fig. 5a,b). The morphology of these fibroblasts was different from that of typical fibroblasts in the middle fibrous layer, as they had abundant cytoplasm, oval nuclei, and a ragged surface. These cell processes extended along the perforating fibre to the bone (Fig. 5b).

### 3D-structure reconstruction

**Figure 6:**
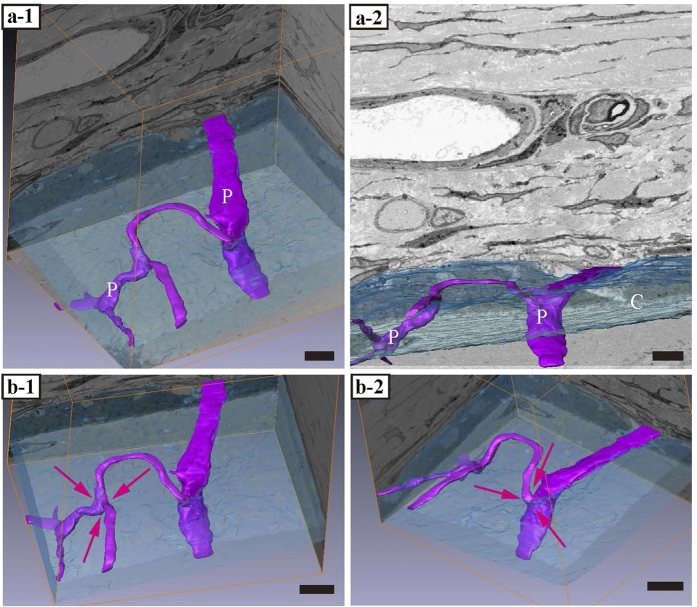
Three-dimensional structure of perforating fibres. **(a-1, a-2**) The perforating fibres are connected to each other and form net-like structures as opposed to nail-like. (**b-1,b-2**) Magenta arrows show the connecting region of the perforating fibres. C, cambial layer (in light blue). P, perforating fibre (in purple). Bar scales, 5 μm for panels a-1 and a-2; 7 μm for panels (**b-1**,**b-2**).

**Figure 7:**
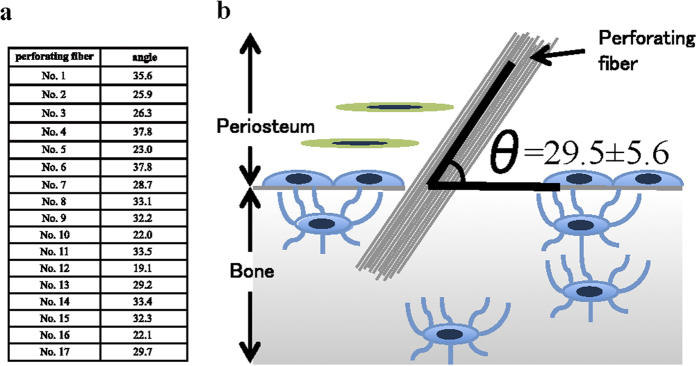
The angle of insertion of the perforating fibre. (**a**) The angles of each perforating fibre (degrees). (**b**) The shape of the perforating angle. The angle is 29.5 ± 5.6 (mean ± standard deviation).

We reconstructed the images based on the FIB/SEM tomography data. Perforating fibres were also connected to each other in the fibrous layer (Fig. 6a-1,a-2, b-1,b-2 [magenta arrows]; Supplementary Movie S1; Supplementary Fig. S1). The angle of the perforating fibre was 29.5 ± 5.6 (mean ± standard deviation) degrees (Fig. 7).

**Figure 8:**
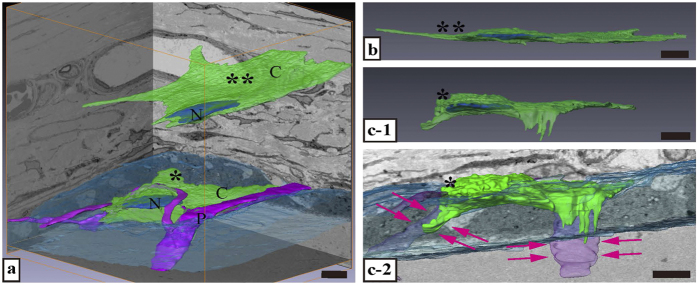
Three-dimensional (3D) structure of fibroblasts in the periosteum. (**a**) Reconstruction images of a typical fibroblast as well as a fibroblast located along the perforating fibre are shown. The asterisks (* and **) correspond to the same as those in Fig. 5. (**b**) 3D reconstruction image of the typical fibroblast. The cytoplasm is scarce and the shape is sheet-like. (**c-1**) 3D reconstruction image of the fibroblast located along the perforating fibre. The cytoplasm is richer than that of the typical fibroblast. (**c-2**) Processes are present in the perforating fibre. The cell processes extend in the same direction as the perforating fibre (magenta arrows). P, perforating fibre. N, cell nucleus. C, cytoplasm. Bar scale: 5 μm in all panels.

The typical fibroblasts in the serial section were reconstructed (Fig. 8a,b). The cytoplasm was scarce, and cells had a sheet-like structure. On 3D ultrastructure imaging, these processes extended horizontally through the fibrous layer.

Fibroblasts located along the lines of the perforating fibre in serial sections were also reconstructed (Fig. 8c-1). Cell processes extended in the direction of the location where the collagen bundle perforated the bone (Fig. 8c-2).

## Discussion

In the present study, we first clarified the ultrastructure of the perforating fibre that helps provide the periosteum’s anchoring strength using FIB/SEM tomography. In previous studies, the perforating fibre in the periosteum was observed with electron microscopy after separating it from the bone surface or treating it with a chemical agent. Hence, it was almost impossible to observe the actual structure of the periosteum. The specimen processing for FIB/SEM as performed in this study is less destructive than that used in previous methods, and is less susceptible to artefacts^23^,^24^,^25^. Therefore, FIB/SEM tomography is presently the preferred method to observe this anatomical structure without being confounded by methodology or artefacts. Although acquisition of data and segmentation of images require time and effort, this technique does have the potential to become a routine method for histological analysis in all tissue types, not only hard tissues, in the future.

Previously, studies of the periosteum focused on investigating cell processes and the perforating fibre, but not the biology of its anchoring properties. Osteoblasts have cell processes that are only connected to osteocytes, and this can increase the anchoring strength. It was considered puzzling that the only components seemingly involved in anchoring the periosteum were osteoblasts and nail-like perforating fibres, which are insufficient to anchor the periosteum and prevent separation by an external force. With light microscopy, we visualized sectioned profile of the perforating fibre and found both ends to be indistinct. With FIB/SEM tomography, however, we discovered that the perforating fibres connect to each other in the fibrous layer of periosteum and form net-like structures. This is the first report to describe these net-like structures of the perforating fibre; these structures evidently provide a stronger anchoring force than that of nail-like structures and may be more effective at resisting mechanical stress.

The angle of the perforating fibre’s attachment to bone was approximately 30 degrees (mean 29.5 ± 5.6). Collagen bundles aside, there are a few reports about the placement angle of mini-screws for suture anchors. Green *et al.* defined the angle of insertion as the angle made between the block and the suture anchor; an angle of insertion at 30 degrees (placement angle of mini-screw) was stronger than that applied at 0 degrees^26^. Our own finding suggests that the perforating fibre provides resistance to pulling in the lateral direction, and prevents separation from the tissue. This also suggests that the net-like structure of the perforating fibre provides resistance to pulling in the upward direction. In this study, the ends of the perforating fibres in the fibrous layer were not observed using FIB/SEM tomography (magnification × 2000). The periosteum widely covers the surface of bones with the exception of the joint. Taken together, our data suggest that the periosteum can exert a strong anchoring force because of its net-like structure and the angle at which the perforating fibres intersect with the bone.

Osteocytes are found in contact with osteoblasts to form lacuno-canalicular networks. These networks sense mechanical stress and modulate several regulatory molecules, including cytokines and transcription factors, and are also involved in bone remodeling^27^,^28^,^29^. Additionally, osteoblast activity is not only affected by osteocyte signalling but also by fibre-to-cell contact^30^. Lee *et al*^31^. reported that, when comparing between three placement mini-screw angles (30, 60, and 90 degrees), mini-screws with a placement angle of 30 and 60 degrees showed a significant increase in maximum stress compared to those at a 90 degrees angle; moreover, a more dramatic increase in maximum stress was noted at 30 degrees than at 60 and 90 degrees. Since we showed that the angle of the perforating fibre was about 30 degrees, these data together suggest that perforating fibres are able to transmit mechanical stress to the bone effectively.

Several types of cells exist in the periosteum, including fibroblasts, osteoblasts, mesenchymal stem cells, and pericytes^11^,^32^. Fibroblasts play several roles in central and peripheral immunological tolerance, wound healing and tissue repair, fibrosis, tumour survival, and metastases^33^,^34^,^35^,^36^. The term ‘fibroblasts’ can apply to a variety of cell populations^37^. In this study, fibroblasts in the periosteum were found to be divided into two morphological types that occurred in different layers. One type exhibited a sheet-like shape in the fibrous layer, and the other produced cell processes extending into the perforating fibres. Fibroblasts that form a collage in the fibrous layer are traditionally called spindle-shaped cells in a single section^37^. However, the fibroblasts that were attached along the collagen bundle that continued to the perforating fibre differ in shape from traditional spindle fibroblasts; the ends of the cellular processes extend to perforating fibres located inside the bone matrix. This strongly suggests that these particular cells were involved in synthesizing the perforating fibre.

We revealed previously unknown structural aspects of the periosteum in this study. These structures enable the periosteum to attach to the bone, function as a cellular source, and transmit mechanical stress. Current therapies tend to only focus on the regeneration of bone material, and various bone regeneration tools such as artificial bone and titanium mesh have been developed to that end. However, effective and reliable therapies developed to date are limited because biological cooperation with surrounding tissues and other organs is essential to achieve proper organ function. We posit that regenerating the periosteum is necessary to maintain a cellular source as well as to facilitate bone regeneration. Functionally, the periosteum requires attachment to the bone, and this by extension is necessary to regenerate the perforating fibre and the management of soft tissue that is required for bone regeneration. Thus, our future challenges are to better understand the role of the periosteum and to develop therapies that promote its regeneration, including that of the perforating fibre.

The present study had several limitations. First, these specimens were obtained from rodents. To attain a wider understanding of the ultrastructure of the periosteum, it is necessary to analyse various species with FIB/SEM tomography. However, rat bones closely resemble human bones, and are frequently used to study hard tissue. Second, our sample size was limited to 100 × 100 × 100 μm using FIB/SEM. To better study the entire ultrastructure of the periosteum, analysis of a wider range of tissue samples and more detailed observation of the distribution of the net-like structure are required. Third, we use the calvarium, which is a flat bone, as the basis of our study; we intend to compare the periosteum and net-like structures between calvaria and long bones in a future study. Finally, we only used 15-week-old Sprague-Dawley rats; however, the structure of the periosteum changes with age^11^,^32^, and it is necessary be cognizant of this fact when performing such studies.

In conclusion, we are the first to observe the 3D-ultrastructure of the periosteum using FIB/SEM tomography. Accordingly, we revealed a net-like structure and also determined the angle between the perforating fibre and the bone. Furthermore, we gained a deeper understanding of the anchoring mechanism of the periosteum and its role as a cellular source and mechanical stress transmitter. These novel findings ought to contribute to developing more effective therapies, including those involving bone regeneration.

## Additional Information

**How to cite this article**: Hirashima, S. *et al.* Anchoring structure of the calvarial periosteum revealed by focused ion beam/scanning electron microscope tomography. *Sci. Rep.* **5**, 17511; doi: 10.1038/srep17511 (2015).

## Supplementary Material

Supplementary Information

Supplementary Movie S1
