# Supplementary material for: Anchoring structure of the calvarial periosteum revealed by focused ion beam/scanning electron microscope tomography
Source: Sci Rep. 2015 Dec 2;5:17511. doi: 10.1038/srep17511 (PMC4667224; doi:10.1038/srep17511)
Supplement: Supplementary Information [file srep17511-s1.pdf]

__**Supplementary information**__

Anchoring structure of the calvarial periosteum revealed by focused ion beam/scanning electron microscope tomography

Shingo Hirashima, Keisuke Ohta, Tomonoshin Kanazawa, Kei-ichiro Uemura, Akinobu Togo, Munetake Yoshitomi, Satoko Okayama, Jingo Kusukawa, and Kei-ichiro Nakamura

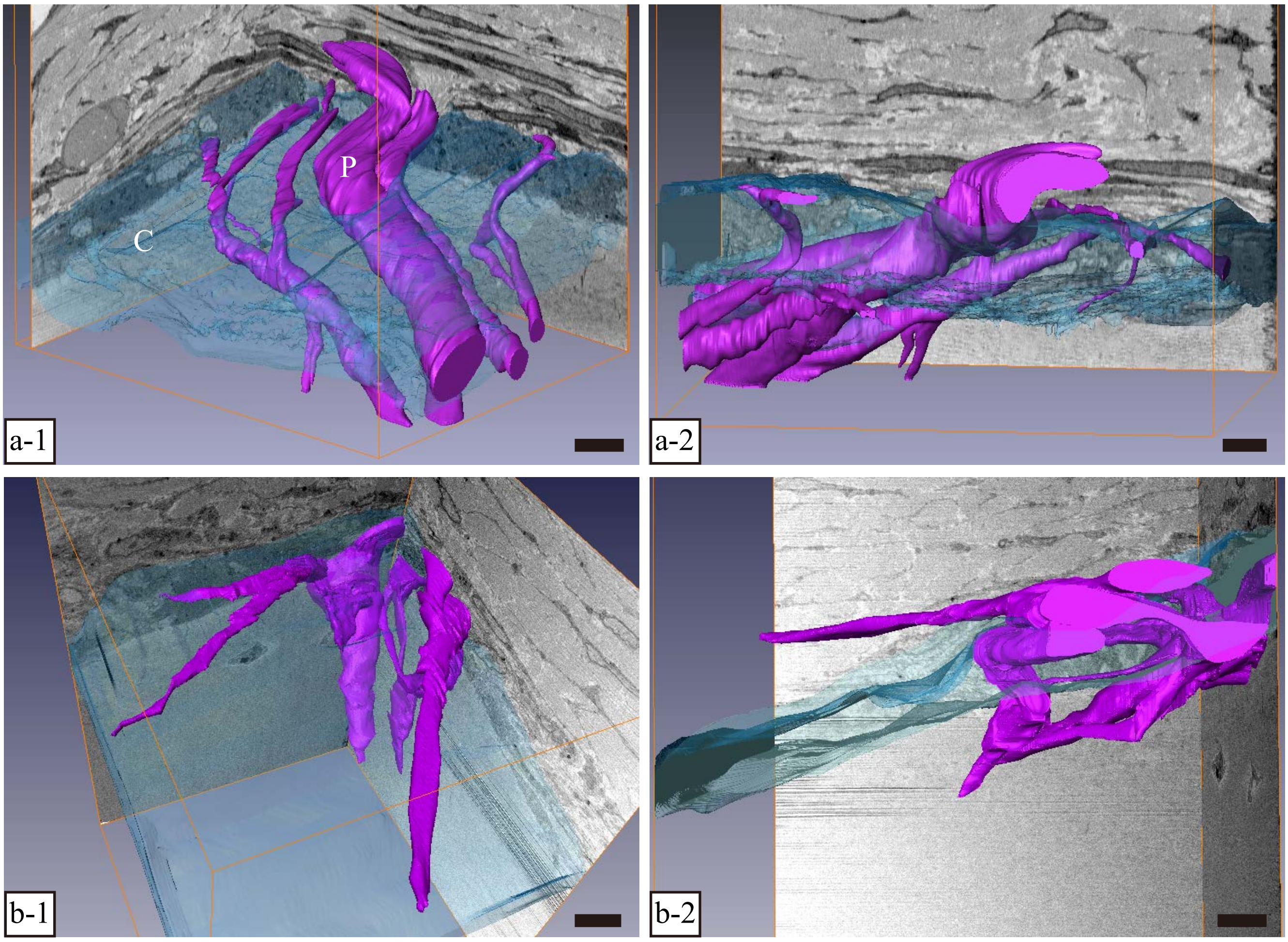

Supplementary Figure 1: Three-dimensional structure of perforating fibres.
The net-like structures of perforating fibres in samples obtained from another rat are shown. C, cambial layer (light blue). P, perforating fibre (purple). Bar scales, 6 μm for panels a-1 and b-1; 5 μm for panels a-2 and b-2.

Supplementary Movie S1: Animation of three-dimensional structure of perforating fibre.
This reconstructed animated sequence is based on the segmented image stack obtained using FIB/SEM tomography, and corresponds to Figures 3, 4, 5, 6, and 8. Perforating fibres (purple) are connected to each other in the fibrous layer. The cambial layer is coloured in light blue, the cytoplasm of the fibroblasts in green, and the nucleus in dark blue.
